# Investigating the Medicinal Potential of *Lavatera cashmeriana* Leaf Extract: Phytochemical Profiling and In Vitro Evaluation of Antimicrobial, Antioxidant, and Anticancer Activities

**DOI:** 10.1155/2024/5301687

**Published:** 2024-08-24

**Authors:** Mohmmad Ashaq Sofi, Mohd Abass Sofi, Anima Nanda, Kasi Thiruvengadam, B. K. Nayak

**Affiliations:** ^1^ Department of Biomedical Engineering Sathyabama Institute of Science & Technology, Chennai 600119, Tamil Nadu, India; ^2^ Department of Chemistry Sathyabama Institute of Science & Technology, Chennai 600119, Tamil Nadu, India; ^3^ Biocontrol and Microbial Metabolites Lab Centre for Advanced Studies in Botany University of Madras Guindy Campus, Chennai, India; ^4^ Department of Botany K. M. Govt. Institute for Postgraduate Studies and Research (Autonomous), Puducherry 605008, India

## Abstract

This study investigated the medicinal potential of *Lavatera cashmeriana*, a plant traditionally known for its therapeutic properties. The aim was to identify the phytocompounds in *L. cashmeriana* leaf extract and evaluate its antibacterial, antioxidant, and anticancer effects. Gas chromatography-mass spectrometry analysis was employed to characterize the phytochemical composition of the ethanol extract derived from *L. cashmeriana* leaves. The antimicrobial potential was assessed through the well diffusion technique, targeting *Escherichia coli*, *Enterococcus faecalis*, *Pseudomonas aeruginosa*, *Staphylococcus aureus*, and *Candida albicans*. The 2,2-diphenyl-1-picrylhydrazyl assay was conducted to assess antioxidant capabilities, while cytotoxicity against the A549 cancer cell line was determined via the MTT assay. GC-MS analysis identified ten different compounds, with phytol, 1-Eicosanol, and 2,6,10-trimethyl,14-ethylene-14-pentadecne being the most prevalent. The extract exhibited notable antimicrobial efficacy against all bacteria with MIC values ranging from 62.5 to 250 *µ*g/mL. However, *C. albicans* did not respond. The extract exhibited antioxidative properties with an IC_50_ value of 86 *µ*g/mL and cytotoxicity with an IC_50_ value of 69.95 *µ*g/mL against the A549 cancer cell line. The results derived from this study supported the historical use of *L. cashmeriana* as a medicinal plant and suggested that it can potentially treat a wide range of medical ailments. The identified phytocompounds and the demonstrated antibacterial, antioxidant, and anticancer effects provide scientific evidence for its medicinal properties. However, further investigations are needed to fully understand its safety profile, efficacy, and mechanism of action before recommending it for therapeutic purposes.

## 1. Introduction

Plants have long been utilized in the treatment and alleviation of numerous illnesses. Despite the efficacy of synthetic drugs in addressing various diseases, herbal remedies are often preferred due to their fewer side effects and greater accessibility [1]. Plants contain secondary metabolites that exhibit diverse biological activities, such as antidiabetic, anticancer, antibacterial, anti-inflammatory, and antioxidant properties [2]. Gaining insight into the chemical makeup of plants is of utmost importance, as this knowledge can contribute to developing novel bioactive compounds tailored to target specific diseases [3]. Phytochemicals derived from medicinal plants are crucial as primary ingredients in the development and discovery of pharmaceuticals [4]. The primary healthcare needs of more than 80% of the global population are met through the use of plant-based remedies, as stated by the WHO [5].

Antimicrobial drug resistance has been a global concern in recent years since resistant microbes cause many health issues due to their genetic plasticity [6]. The most common pathogenic bacteria are *S. aureus*, *P. aeruginosa*, *K. pneumoniae*, *S. typhimurium*, *S. epidermidis*, *E. coli*, and *C. albicans*, which have been linked to antimicrobial resistance [7].

Research on relationships between plant-based antioxidants and the prevention of noncommunicable diseases, like diabetes, cancer, and cardiovascular, has increased sharply in recent years [8]. Epidemiological and in vitro investigations provide strong evidence supporting the potential use of plants containing antioxidant phytochemicals to treat various ailments. Phytochemicals, mainly phenolic compounds, have been shown to reduce oxidative stress and inhibit macromolecular oxidation, thereby decreasing the risk of degenerative diseases [9]. Consequently, there is a growing interest in identifying plant extracts that exhibit effective and nontoxic properties.

In the present era, cancer continues to be one of the most formidable illnesses, posing an imminent danger to human life. Numerous internal and external factors contribute to the onset of this disease. Despite recent strides in the development of novel anticancer medications, cancer remains a leading cause of mortality on a global scale [10]. The emergence of resistance to chemotherapy compounds further complicates the effectiveness of cancer treatment, serving as a significant obstacle. Consequently, the exploration and identification of fresh, efficacious anticancer drugs to counteract this resistance have become imperative challenges [11]. Over the past few decades, natural products have significantly contributed in the development of chemotherapeutic drugs. A noteworthy proportion of successful cancer treatments owe their success to compounds sourced from nature [10]. Approximately, 60% of the drugs presently employed in cancer treatment trace their origins back to natural sources [12].

*Lavatera cashmeriana* is an important medicinal herb of the Kashmir Himalayas that belongs to the Malvaceae family. Despite being native to the Kashmir valley, it is now widespread throughout the western Himalayas, from Pakistan to Uttar Pradesh and Uttaranchal. *L. cashmeriana* roots have been traditionally employed to address various health issues, including gastrointestinal problems, kidney stones, and dandruff. They serve as a laxative and are believed to stimulate hair growth when applied to the scalp. Traditionally, the roots of *L. cashmeriana* are used to treat gastrointestinal diseases, renal colic, and dandruff. The root decoction of *L. cashmeriana* is used as a laxative and is also said to promote hair growth when applied to the scalp. Its flowers are used to treat common colds and mumps, and its seeds are used as an antiseptic [13, 14]. The leaves and petals have been used to treat skin irritation in pregnant women, urinary issues, and as an antiseptic [15–17]. Recent research on this species has revealed some biological activities, including antilipoxygenase activity [18] as a protease inhibitor [19]. Crude extracts of medicinal plants are believed to possess higher biological activity than isolated phytocompounds due to their synergistic effects [20]. The synthesis of these secondary metabolites is genetically controlled and is strongly influenced by various biotic and abiotic stresses. Various environmental factors, such as precipitation, mean temperature, soil characteristics, and radiation, change with altitude [21, 22]. The present study aims to identify chemical constituents and assess the antimicrobial, anticancer, and antioxidant properties of the ethanolic leaf extract of *L. cashmeriana.*

## 2. Materials and Methods

### 2.1. Plant Collection and Processing

**Figure 1:**
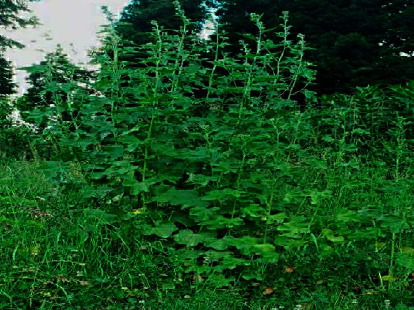
*Lavatera cashmeriana* plant.

**Figure 2:**
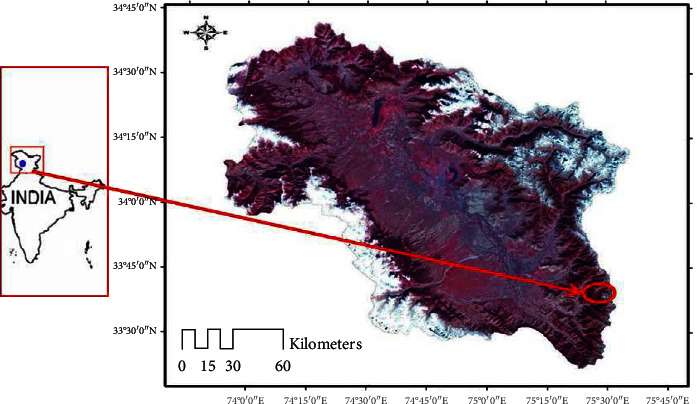
Site at Anantnag Daksum, Kashmir.

The plant depicted in Figure 1, *L. cashmeriana*, was procured from Daksum Anantnag, Jammu and Kashmir, India, in June 2019. This region is situated at an elevation of 2,438 meters (Figure 2). The plant was identified and authenticated at the Kashmir University's Center for Biodiversity and Taxonomy (CBT-botany) under voucher 3070-(KASH) Herbarium. The plant material (leaves) was subjected to a shade-drying process under hygienic conditions for a minimum of 15 days to facilitate further processing. Subsequently, an electrical grinder was used to crush the dried leaves into a coarse powder form. The resulting powder was then packaged carefully to maintain its quality and purity for future use in research.

### 2.2. Extraction Process

The extraction of the plant material was performed using a simple maceration process [1]. A total of 10 grams of coarse powder from the plant material was combined with 200 mL of ethanol, of a desired quality grade, in a flask. The mixture was then placed on a shaker and underwent extraction for 24 h at room temperature. After 24 h, the reaction mixture was filtered using Whatman filter paper No. 1. The obtained filtrate was further processed through solvent evaporation to get a more concentrated extract. This process was performed thrice to ensure the sample's sufficient quantity and quality for subsequent analysis.

(1) $$\begin{array}{c} \text{Total extraction yield }\%=\left(\frac{\text{mass of the sample}}{\text{mass of the extract}}\;\right)\times 100. \end{array}$$

## 3. GC-MS Analysis

Phytochemical profiling analysis was conducted using the GC-MS Shimadzu-QP2010. The extract was introduced to a capillary column through split mode injection with split ratio of 1 : 2. The carrier gas used was helium, and the flow rate was set at 1 mL per minute. The analysis spanned 50 minutes. Initially, the column oven was set at 80°C. Subsequently, the column temperature was gradually increased by 3°C per minute until it reached 200°C. Following this, the temperature was further elevated to 260°C at a rate of 10°C per minute and held steady for 5 minutes at that level [23].

## 4. List of Microbial Species

Table 1 represents the microbial species employed in the antimicrobial assay screening.

## 5. Antimicrobial Assay

We evaluated the antimicrobial properties of the methanolic leaf extract from *L. cashmeriana* against different microbes (Table 1) using the well diffusion method. Initially, sterile Muller Hinton Agar was poured into Petri dishes and allowed to solidify. Subsequently, test microorganisms were uniformly spread over the agar surface at densities ranging from 10^4^ to 10^6^ CFU/mL. Wells were created on the agar surface using a cork borer, and four wells were filled with varying concentrations of the *L. cashmeriana* extract ranging from 25 to 100 *μ*L, each from a 10 mg/mL stock solution. The fifth and sixth wells served as the positive and negative controls, respectively. Following incubation at 37°C for 16-18 h, zones of inhibition were measured around the wells.

### 5.1. Determination of the MIC

The procedure was executed within 96-well plates, following the methodology outlined by Gabrielson et al. [24]. The initial ten wells of the plate accommodated diverse concentrations of *L. cashmeriana* leaf extract, ranging from 500 to 0.9 *µ*g/mL. Once the wells were loaded with the extract, 5 *µ*L of 12 h old microbial culture was introduced to each well. Subsequently, the plates were positioned within an incubator set to 37°C, where they remained for a duration of 16-18 h. Subsequent to the incubation, 10 *µ*L of a 5 mg/mL MTT solution was incorporated into each well. MTT, a yellow dye, is transformed into a purple formazan product exclusively by viable cells. Following 2 h incubation alongside MTT, 100 *µ*L of DMSO was introduced into each well to facilitate the dissolution of the formazan product. The visual observation of the transition in color, shifting from yellow to purple, enabled the determination of the MIC, an indicator of the lowest extract concentration entirely halting microbial proliferation.

## 6. Antioxidant Activity

The radical scavenging activity (RSA) of the sample was evaluated using the DPPH assay, following the methodology outlined by Chang et al. [25] with minor adjustments. In this procedure, a 0.1 mM ethanolic DPPH solution (2960 *µ*L) was mixed with varying concentrations of the plant extract (20, 40, 60, 80, and 100 *µ*g), alongside a blank solution composed of 0.1 mM ethanolic DPPH solution (40 *µ*L) mixed with distilled water. The solution was vigorously mixed using a vortex mixer and afterwards placed in a dark environment at room temperature for a period of 20 minutes. Subsequently, the absorption of the mixtures was recorded at 517 nm. As a reference compound, BHT was employed. The RSA was computed using the subsequent formula: RSA (%) = (absorbance control − absorbance sample) × 100.

## 7. Cell Culture

The A549 human lung cancer cell line was obtained from NCCS Pune, India. These cells were cultured in DMEM (Sigma-Aldrich, United States, D1152) supplemented with 10% FBS (Gibco, United States, 10270106) and 1% antibiotic-antimycotic solution. They were maintained in an incubator at 37°C with 5% CO_2_. The cells were allowed to grow until they reached 70–80% confluency, indicating that they had formed a monolayer on the surface of the culture dish. Trypsinization was performed using 1× trypsin EDTA (Gibco, Catalogue: 25200-056) solution to propagate the cells further. Following trypsinization, the A549 cells were seeded into a 96-well plate and given 21-24 h to attach to the surface.

### 7.1. MTT Assay

In this experiment, viable cells were cultured in a 96-well plate, with each well containing a density of 6,000 cells. Extracts were prepared at various concentrations (6.5, 12.5, 25, 75, 100, and 125 *μ*g/mL) using a culture medium. This treatment was administered over 24 and 48 h. Following the treatment period, the medium containing the extract was carefully removed and substituted with a new serum-free medium. After incubation, the MTT reagent was introduced into each well and allowed to incubate for another 4 h. The formation of formazan crystals, indicating viable cells, was facilitated by the MTT reagent. Subsequently, these crystals were solubilized using DMSO, and the absorbance was determined at 570 nm [26]. The % of cell inhibition was calculated employing the following equation:

(2) $$\begin{array}{c} \%\text{ of viability}=\left(\frac{\text{absorbance of control cells}}{\text{absorbance of treated cells}}\right)\times 100. \end{array}$$

### 7.2. Morphological Analysis

Cells were seeded onto glass coverslips in a 6-wellplate and allowed to grow until they reached a healthy morphology and achieved 60–80% confluence. Once this stage was reached, the cells were exposed to specific IC_50_ concentrations of *L. cashmeriana* leaf extract and incubated for 24 h. Upon completion of the experiment, the cells were rinsed twice with PBS. Morphological analysis was then performed using an inverted microscope (Motic type 101M).

## 8. Statistical Analysis

The experiments were carried out, and the data were analyzed to compute the mean ± standard deviation and IC_50_ values for both DPPH and MTT assays using Microsoft Excel 2019.

## 9. Results and Discussion

The percentage of plant extract obtained using ethanol as a solvent was assessed, and the outcomes are presented in Table 2.

**Figure 3:**
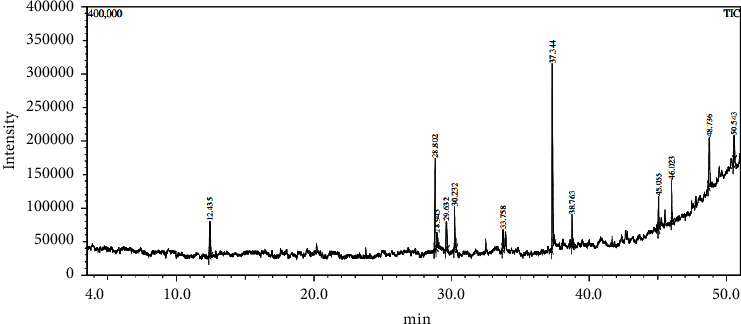
Chromatogram of *L. cashmeriana* ethanolic leaf extract.

The GC-MS study of the *L. cashmeriana* ethanolic leaf extract resulted in the identification of ten compounds (Figure 3 and Table 3).

Of these phytocompounds, the most prevailing compounds were phytol, 2,6,10-trimethyl,14-ethylene-14-pentadecane, and 1-Eicosanol. Phytol is the most prevalent and identified compound in the leaf extract of *L. cashmeriana*. Phytol has been investigated for its health benefits, including its ability to decrease blood sugar levels, reduce inflammation, and improve liver function. It is also commonly used in the fragrance and flavor industry and the production of vitamins and other supplements. It has also been shown to have potential as an antimicrobial, anticancer agent, as it has been found to induce apoptosis in cancer cells [27].

**Figure 4:**
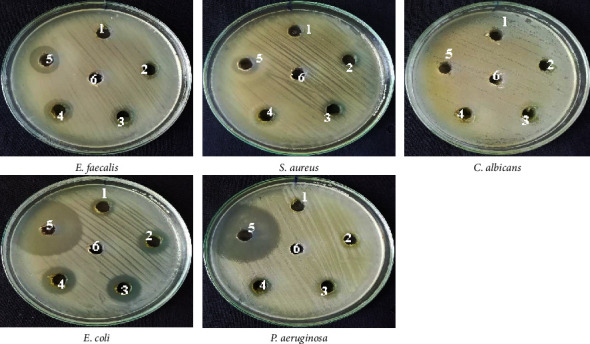
The well diffusion method for determining the antibacterial property of ethanolic leaf extract of *L. cashmeriana*. The numbers given to each Petri dish denote the wells that contain various concentrations of *L. cashmeriana* leaf extract, standard medications at 25 *µ*L (1 mg/mL), and negative control. The specific concentrations are as follows: 1 : 25 *µ*L; 2 : 50 *µ*L; 3 : 75 *µ*L; 4 : 100 *µ*L; 5 : 25 *µ*L. The positive control consists of Ampilox for Gram-positive bacteria, Fluconazole for fungi, and Ciprofloxacin for Gram-negative bacteria (labeled as 6). In addition, DMSO: negative control.

The emergence of antibiotic resistance and new pathogens has made treating bacterial infections more challenging. Therefore, finding new and effective bioactive agents to combat microbial infections is urgently needed. Despite the availability of numerous drugs for treating microbial infections, their adverse side effects can limit their usage in specific populations, making it difficult to treat microbial infections, especially in vulnerable populations [28, 29]. Plant extracts are active against various pathogenic microorganisms, including *S. typhi*, *E. coli*, *P. aeruginosa*, *C. albicans, S. aureus*, and *B. subtilis*, among others. As a result, researchers continue to explore the potential of plant extracts as a source of new and effective bioactive agents to treat bacterial infections [30]. *L. cashmeriana*, a medicinal plant commonly used in traditional remedies by local inhabitants, was tested against different microbial strains to evaluate and examine its antimicrobial capabilities. The crude ethanolic leaf extract of *L. cashmeriana* demonstrated the highest antimicrobial activity against *E. coli* 19 ± 0.57 mm, *E. faecalis* 16 ± 0.63 mm, followed by *P. aeruginosa* 14 ± 0.45 mm and *S. aureus* 11 ± 0.45 mm with MIC values ranging from 62.5 to 250 *μ*g/mL. However, the extract did not exhibit antifungal activity against *C. albicans*, indicating that it may not be effective against infections caused by this microbe, as shown in Figure 4 and Table 4, with zones of inhibition and MIC values, respectively. The results from this research align with findings from previous studies on the *Lavatera* genus and Malvaceae family, which also demonstrated significant antimicrobial activity against various microorganisms [31–34]. Skalicka-Woźniak et al. [31] found that the chloroform extract of *L. trimestris* exhibited considerable antibacterial activity against *S. aureus*, *E. coli*, and *P. aeruginosa*. Similarly, Sofi et al. [34] found strong activity against *E. coli, P. aeruginosa*, *S. aureus*, and *E. faecalis* in the methanolic root extract of *L. cashmeriana*. Despite the promising antibacterial potential of *L. cashmeriana*, its leaf extract did not exhibit activity against *C. albicans*. This observation was consistent with other studies on *L. cashmeriana* methanolic root, ethanolic extract from leaves of *Lavatera arborea* L, and ethanolic extract of aerial parts of *Sida acuta*, which also demonstrated no activity against *C. albicans* [34, 35]. These results highlight the need for further research to identify plant extracts with both antibacterial and antifungal properties to combat a broader range of infections effectively.

The findings of this study are encouraging, as the examined microbes commonly cause community- and hospital-acquired infections. The growing antibiotic resistance underscores the need for new drugs with novel targets. The antimicrobial activity of *L. cashmeriana* is relevant in this perspective since the MIC values of the active extract in the present investigation are below 1 mg/mL, which may be regarded as significant in light of the discovery of potent antimicrobials from plants.

**Figure 5:**
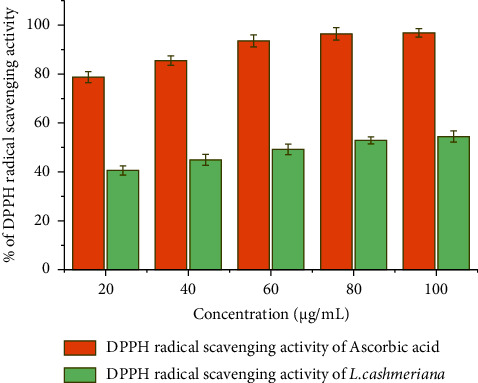
The capacity of ethanolic leaf extract of *L. cashmeriana* and ascorbic acid to neutralize DPPH radicals is depicted at various concentrations. Each value displayed represents the mean ± SD, based on three repetitions (*n* = 3).

The generation of free radicals causes several reactions that may harm tissues, cell function, and macromolecules [36]. Oxidative stress has been linked to several pathological disorders, including aging, atherosclerosis, diabetes, and cancer [37]. Nonetheless, antioxidants provide a promising defense against oxidative stress-related diseases by slowing the rate of oxidation or inhibiting the propagation of free radical production, resulting in anticancer, anti-aging, and anti-inflammatory properties [38]. The antioxidant property of crude extract was assessed using the DPPH radical scavenging test in this study. The findings of this assay are shown in Figure 5, which compared the DPPH radical scavenging activity of *L. cashmeriana* leaf extract to that of ascorbic acid. Although the standard antioxidant had more scavenging activity than the extract at all tested concentrations, the extract nonetheless demonstrated considerable free radical scavenging activity. One of the methods through which *L. cashmeriana* is beneficial as traditional medicine is its ability to scavenge free radicals. Consumption of *L. cashmeriana* leaves may help in preventing oxidative stress-related degenerative illnesses. The antioxidant activity of *L. cashmeriana* might be due to the presence of flavonoid and phenolic compounds. Phenolic and flavonoid compounds have been linked to antioxidative activity in biological systems, serving as scavengers of free radicals [39]. Consumption of *L. cashmeriana* leaves may help in preventing oxidative stress-related degenerative illnesses.

Cancer has become a significant cause of mortality in underdeveloped nations, surpassing AIDS, TB, and malaria combined. Lung cancer is a particularly prevalent type, leading to a large number of deaths worldwide [40, 41]. Although synthetic chemistry has recently dominated drug discovery and production, plant-derived medications have significantly impacted the antitumor field. In light of this, the potential of bioactive plants and their extracts to provide innovative disease treatment and prevention solutions remains immense. Some of the notable plant-derived agents that have improved chemotherapy for various types of cancer include taxol, vinblastine, camptothecin, and vincristine [42]. The vast potential of plants to produce chemicals attracts researchers seeking new and novel chemotherapeutic agents [43]. As a result, exploring anticancer chemicals in plants and traditional foods is a promising and viable cancer prevention strategy [44]. Plant-based compounds with anticancer properties can be found in several categories, such as terpenoids, phenylpropanoids, and alkaloids [45]. Continued research into these compounds holds excellent potential for the development of new, effective treatments for cancer, especially in underdeveloped nations where access to conventional therapies might be limited.

Harnessing the power of plants and their extracts can revolutionize cancer treatment and prevention, offering hope to millions of patients worldwide. Many plant-derived medicines are effective in treating different types of cancer. Plant-derived medicines often have advantages, including better patient tolerance and fewer negative side effects in comparison to their synthetic counterparts. In addition, the development of drug resistance is less common with plant-derived medications [43, 46].

**Figure 6:**
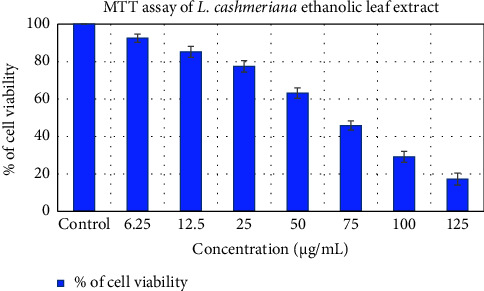
MTT assay of A549 cells treated with *L. cashmeriana* ethanolic leaf extract. The error bars depict the standard deviation from the mean. *n* = 3.

In the past, extracts and isolated components derived from plants belonging to the Malvaceae family have shown noteworthy effectiveness in inhibiting the growth of cancerous cells. This current investigation focused on evaluating the impact of an ethanol-based leaf extract from *L. cashmeriana* on A549 cells. The extract was found to be cytotoxic to A549 cells, with an IC_50_ value of 69.95 *µ*g/mL compared to control (Figure 6). The cytotoxicity increased with an increase in extract concentration, as observed in the MTT results. The study suggests that the ethanolic extract of *L. cashmeriana* leaves showed strong anticancer activity against A549 cells in a dose-dependent manner. Thus, it has the potential for use in cancer prevention and chemotherapy. Earlier research has documented the in vitro potential of extracts from the same family to exhibit antilung cancer properties. Rakshanda et al. [47] studied the impact of *L. cashmeriana* protease inhibitors (LC-pi) isolated from seed on A549 cell proliferation. LC-pi I and II inhibited A549 cell growth. These compounds showed concentration-dependent inhibitory actions. After 48 h, IC_50_ values for LC-pi I and II were 54 *μ*g/mL and 38 *μ*g/mL, respectively. In related studies, the different extracts of *Hibiscus sabdariffa* Linn demonstrated anticancer activities against A549 cells, with the ethanolic extract being the most potent (IC_50_ value of 374.01 *μ*g/mL) [48]. Similarly, the ethanolic leaf extract of *Abutilon indicum* L exhibited antiproliferative effects against the A549 cells, with an IC_50_ value of 85.2 *µ*g/mL [49]. In another study, the antiproliferative efficacy of the methanolic extract of different parts of *Grewia orbiculata* plant was assessed on the A549 lung cancer cell line, revealing an IC_50_ value of 98.73 *µ*g/mL [50]. Furthermore, the ethanolic leaf extract derived from *A. indicum* demonstrated notable antiproliferative effects against the A549 cell line, achieving a substantial cell inhibition rate of 72.1% at a concentration of 200 *μ*g/mL [51]. Our findings align with these earlier studies, suggesting that the ethanolic extract could be a promising candidate for further research and development of anti lung cancer therapies. While preliminary studies have shown promising anticancer properties of *L. cashmeriana*, further research is essential to validate these findings and explore the underlying molecular mechanisms responsible for its anticancer effects.

**Figure 7:**
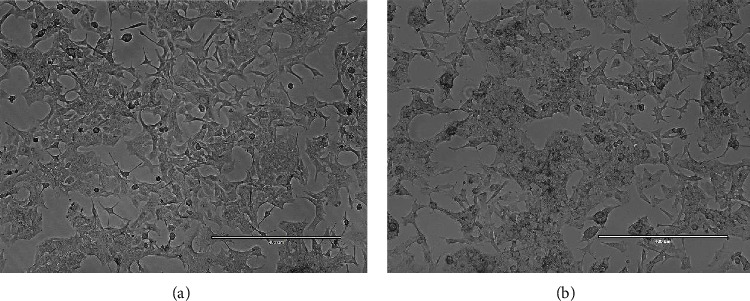
(a, b) The morphology of A549 cells in two groups: the control group (a) and the group treated with the IC_50_ value (b). The cells treated with the IC_50_ value (b) demonstrate noticeable characteristics such as shrinkage and deformed membranes.

The impact of *L. cashmeriana* extract on the structure of A549 cells was investigated. In the control group, cells displayed regular growth patterns with distinct nuclei. Furthermore, control cells exhibited excellent adherence in a single layer, whereas the treated cells displayed atypical structure and distorted cell membranes, as evidenced by microscopic analysis (Figures 7(a) and 7(b)). These treated cells demonstrated signs of shrinkage and distorted membranes, consistent with the attributes of cells undergoing apoptosis, as previously documented by researchers [52, 53].

The antimicrobial, antioxidant, and antiproliferative properties of the ethanolic leaf extract of *L. cashmeriana* can be attributed to bioactive compounds. Some of the major compounds identified in our study, phytol, biphenyl, 1-Eicosanol, and 1,2-Benzenedicarboxylic acid 3-nitro, have been shown to exhibit antimicrobial and antioxidant properties [27, 54–57]. Phytol, 2,6,10-trimethyl-14-ethylene-14-pentadecene, and butyl 9,12,15-octadecatrienoate are reported to display antiproliferative properties [58–60].

Phytol, the predominant compound in the ethanolic extract of *L. cashmeriana*, has been shown to hinder angiogenesis and enhance cell death in A549 cells by altering the mitochondrial membrane potential [61, 62]. In a separate investigation, it was observed that phytol triggered both apoptosis and protective autophagy in AGS cells. The autophagy inhibitor CQ intensified phytol's inhibitory impact on cell proliferation and facilitated apoptosis in gastric cancer cells [63]. Likewise, phytol induces apoptotic alterations and generates ROS in A549 cells by activating TRAIL, FAS, and TNF-*α* receptors, along with caspase 9 and 3 [64]. Phytol has been identified as an inducer of ROS-mediated apoptosis in *S. pombe*. In addition, it has been confirmed that phytol effectively inhibits cellular senescence caused by oxidative stress from H_2_O_2_ [65, 66].

Thorough investigations of the above analytical compounds present in this plant reveal tremendous medical significance, owing to the presence of varied phytoconstituents, which can treat various diseases and potentially serve as precursor compounds for numerous medicinal and other applications.

## 10. Conclusion

In conclusion, this research article highlighted the medicinal potential of *L. cashmeriana*, a plant traditionally used for its therapeutic properties. The study identified ten compounds in the ethanol extract of *L. cashmeriana* leaves, with phytol, 1-Eicosanol, 2,6,10-trimethyl, and 14-ethylene-14-pentadecane being the most prevalent. The extract exhibited antimicrobial efficacy against *E. coli*, *E. faecalis*, *P. aeruginosa*, and *S. aureus*. However, it did not affect *C. albicans*. In addition, the extract showed antioxidant properties and a strong cytotoxic effect on the A549 cancer cell line. These positive outcomes necessitate further research to elucidate the mechanisms behind these effects, including the safety and efficacy of the extract in vivo, as well as the exploration of novel therapeutic agents from its active components for the treatment of infectious diseases and cancer.

## Figures and Tables

**Table 1:** The microbial species employed in the antimicrobial assay screening.

Test organism	Source	Microbial type
*E. faecalis*	ATCC 25923	Gram positive
*S. aureus*	ATCC 29212	Gram positive
*E. coli*	ATCC 11229	Gram negative
*P. aeruginosa*	ATCC 15442	Gram negative
*C. albicans*	ATCC 10231	Fungus

**Table 2:** The yield of extraction of *L. cashmeriana* in ethanol.

Species	Mass of the dried leaf (g)	Mass of the extract (g) yield %
*L. cashmeriana*	10	1.421 (14.21%)

**Table 3:** Phytocompounds reported in the ethanolic leaf extract of *L. cashmeriana* by GC-MS.

Peak#	R. time	Area%	Name	Molecular formula	Molecular weight
1	12.435	5.44	Biphenyl	C_12_H_10_	154
2	28.802	16.69	2,6,10-Trimethyl,14-ethylene-14-pentadecne	C_20_H_38_	278
3	28.945	2.93	2-Pentadecanone,6,10,14-trimethyl-	C_18_H_36_O	268
4	29.632	4.62	3,7,11,15-Tetramethyl-2-hexadecen-1-ol	C_20_H_40_O	296
5	30.232	6.92	2,6,10-Trimethyl,14-ethylene-14-pentadecne	C_20_H_38_	278
6	33.758	2.95	2A-Hydroxymanoyloxide	C_20_H_34_O_2_	306
7	37.344	33.56	Phytol	C_20_H_40_O	296
8	38.763	4.61	Ethyl(9Z,12Z)-9,12-octadecadienoate	C_20_H_36_O_2_	308
9	45.055	4.26	Butyl9,12,15-octadecatrienoate	C_22_H_38_O_2_	334
10	46.023	5.10	1,2-Benzenedicarboxylic acid, 3-nitro-	C_24_H_38_O_4_	390
11	48.736	8.93	1-Eicosanol	C_20_H_42_O	298
12	50.543	4.00	2,6,10,14,18-Pentamethyl 2,6,10,14,18-Icosapentaene	C_25_H_42_	342
		100.00			

**Table 4:** The zone of inhibition of the ethanolic leaf extract of *L. cashmeriana* against microorganisms.

Zone of inhibition (mm)						
Plant extract stock solution 10 mg/mL				1 mg/mL standard drug		
				Ampilox/ciprofloxacin, fluconazole		
Microbe names	25 *µ*L	50 *µ*L	75 *µ*L	100 *µ*L	25 *µ*L	DMSO
*E. faecalis*	—	—	12 ± 0.4	16 ± 0.6	17 ± 0.7	—
*S. aureus*	—	—	11 ± 0.6	14 ± 0.4	18 ± 0.8	—
*C. albicans*	—	—	—	—	—	—
*E. coli*	12 ± 0.5	16 ± 0.7	18 ± 0.8	19 ± 0.6	36 ± 0.4	—
*P. aeruginosa*	—	—	12 ± 0.4	14 ± 0.5	34 ± 0.6	—

Gram-positive bacteria		MIC (*µ*g/mL) Gram-negative bacteria		Fungus		

*E. faecalis*	*S. aureus*	*P. aeruginosa*	*E. coli*	*C. albicans*		
125	250	250	62.5	ND		

The symbol — denotes no activity, and the reported value represents the mean ± SD obtained from three repetitions (*n* = 3). ND: not determined, as they did not show antimicrobial activity in the well diffusion assay.

## Data Availability

All relevant data for this research are provided in the manuscript.
